# Challenges to Engage Low-Skilled Adults in Education and Training: An International Perspective

**DOI:** 10.1093/geroni/igab046.1508

**Published:** 2021-12-17

**Authors:** Sydney Shadovitz, Abigail Helsinger, Phyllis Cummins

**Affiliations:** 1 Miami University, Oxford, Ohio, United States; 2 Scripps Gerontology Center, Miami University, Oxford, Ohio, United States

## Abstract

The demand for adult education and training (AET) opportunities throughout the life course is substantial as labor markets often require workers to obtain advanced skills. AET opportunities are more often pursued by high-income and high-skilled workers than low-skilled or low-income workers. With the increased prominence of job automation and technological advances in the workforce, low-skilled workers are at risk for fewer opportunities within the labor market. These factors emphasize the importance of providing learning opportunities throughout the life course. In this mixed-methods study, we analyzed 2012/2014 data from the Program for the International Assessment of Adult Competencies (PIAAC) for the U.S., Canada, the Netherlands, Norway, and Sweden to compare participation rates in non-formal education (NFE) by high and low-skilled adults. Countries were selected based on qualitative findings that inform best practices. Additionally, to gain insights of policies and programs that promote NFE, international key informant interviews (n = 33) were conducted. AET policies and programs, along with barriers such as cost, motivation, and time, were explored with key informants. Findings include (1) aging and skills are negatively correlated in all nations of interest; (2) low-skilled adults are less likely to participate in NFE than their high-skilled counterparts; (3) low-skilled workers in Norway and the Netherlands are more likely to participate in NFE than their U.S. counterparts; and (4) NFE is often more acceptable to low-skilled adults due to previous negative experiences with formal education. Using these findings, we discuss successful AET programs in Nordic countries for overcoming barriers.

